# Primary Cilia and Atherosclerosis

**DOI:** 10.3389/fphys.2021.640774

**Published:** 2021-02-02

**Authors:** Zhi-Mei Wang, Xiao-Fei Gao, Jun-Jie Zhang, Shao-Liang Chen

**Affiliations:** Department of Cardiology, Nanjing First Hospital, Nanjing Medical University, Nanjing, China

**Keywords:** shear stress, endothelial cells, mechanical sensor, primary cilia, axoneme, vesicle trafficking

## Abstract

In artery tree, endothelial function correlates with the distribution of shear stress, a dragging force generated by flowing blood. In laminar shear stress areas, endothelial cells (ECs) are available to prevent atherosclerosis, however, ECs in disturbed shear stress sites are featured with proinflammation and atherogenesis. Basic studies in the shear stress field that focused on the mechanosensors of ECs have attracted the interest of researchers. Among all the known mechanosensors, the primary cilium is distinctive because it is enriched in disturbed shear stress regions and sparse in laminar shear stress areas. The primary cilium, a rod liked micro-organelle, can transmit extracellular mechanical and chemical stimuli into intracellular space. In the cardiovascular system, primary cilia are enriched in disturbed shear stress regions, where blood flow is slow and oscillatory, such as the atrium, downstream of the aortic valve, branches, bifurcations, and inner curves of the artery. However, in the atrioventricular canal and straight vessels, blood flow is laminar, and primary cilia can barely be detected. Primary cilia in the heart cavity prevent ECs from mesenchymal transition and calcification by suppressing transforming growth factor (TGF) signaling. Besides, primary cilia in the vascular endothelium protected ECs against disturbed shear stress-induced cellular damage by triggering Ca^2+^ influx as well as nitric oxide (NO) release. Moreover, primary cilia inhibit the process of atherosclerosis. In the current review, we discussed ciliogenesis, ciliary structure, as well as ciliary distribution, function and the coordinate signal transduction with shear stress in the cardiovascular system.

## Introduction

Blood flow generates various types of forces, such as compression, cyclic strain, pressure, stretch, and shear stress, among which shear stress is the most widely studied (Lee et al., 2015; Lehoux and Jones, 2016). Shear stress, a blood flow-generated dragging force, affects the intima directly. In humans, shear stress ranges from 10 to 70 dynes/cm^2^ in the straight artery; however, in branches, bifurcations, and the inner curves of the artery, flow is disturbed, and the net value of shear force is always less than 4 dynes/cm^2^ (Chiu and Chien, 2011). In laminar shear stress regions, endothelial cells (ECs) maintain antiproliferative, antithrombotic, and anti-inflammatory phenotypes. In contrast, in disturbed flow regions, ECs are identified with accumulated reactive oxygen species (ROS), decreased nitric oxide (NO), and exaggerated inflammation responses (Chiu and Chien, 2011; Hsieh et al., 2014; Lehoux and Jones, 2016; Chistiakov et al., 2017).

The responses of ECs to shear stress are closely linked to intravascular coagulation and fibrinolysis, angiogenesis, and vascular remodeling. They also participate in maintaining vascular homeostasis (Hsieh et al., 2014; Qu et al., 2019). Two distinctive mechanisms are involved in endothelial mechanotransduction. One is termed mechanotransmission, a process in which shear stress is transmitted from continuous blood flow onto the cell surface and then internally within the cell via the cytoskeleton. The other is indirect transduction, in which shear stress is converted into biological stimuli by specialized cellular components termed mechanotransducers. Numerous postulated membrane molecules and microdomains are involved in mechanotransduction, including intercellular tight junctions, ion channels, G protein-coupled receptors, adhesion molecules, cytoskeletons, caveolae, glycocalyx, and primary cilium. Upon stimulation, mechanotransduction sensors and signals and multiple downstream signaling pathways get activated almost simultaneously (Chiu and Chien, 2011; Ando and Yamamoto, 2013). Among all these mechanosensors, the primary cilium is unique because it is enriched in disturbed shear stress regions and can barely be detected in laminar shear stress areas in the cardiovascular tree (Iomini et al., 2004).

The cilium, a rod-like organelle that protrudes into the lumen, is an identified chemical and mechanical sensor that serves as an antenna to amplify and transduce extracellular signaling (Hierck et al., 2008). The ciliary function was not elaborated until it was associated with human disorders such as Bardet-Biedl syndrome and polycystic kidney disease (PKD) (Singla and Reiter, 2006). Primary cilia dysfunction results in a wide range of diseases classified as ciliopathies, including Bardet-Biedl syndrome, Joubert syndrome, Meckel-Gruber syndrome, and nephronophthisis (Reiter and Leroux, 2017; Pala et al., 2018). Identifications of primary cilia in the human cardiovascular system were first described in the 1980s (Haust, 1987; Bystrevskaya et al., 1988). Impaired primary cilia are associated with many vascular diseases, such as hypertension, aneurysm, left ventricular hypertrophy, and mitral valve prolapse (Boucher and Sandford, 2004; Torres et al., 2007). Primary cilia in artery trees are characterized by their accumulation in disturbed shear stress regions where plaques tend to deposit. However, systematic knowledge of primary cilia in atherosclerosis is not well established. This review discusses the ciliogenesis and ciliary structure and the distribution, function, and coordinate signal transduction of primary cilia with atherosclerosis.

## Ciliogenesis

In most cases, a primary cilium is formed at the terminal stage of mitosis after cell polarity is established and can only be detected in the G0/G1 phase (Bystrevskaya et al., 1992). During the initial stage of ciliogenesis, vesicles package the mother centriole and then transfer to the apical plasma membrane. After being ingrained beneath the basal body, the primary cilia elongate and extrude from the cell surface. At the end phase of ciliogenesis, the cellular membrane fuses into the ciliary membrane, the centrioles act as basal bodies, and the spindle fibers act as axonemes (Wheatley et al., 1996; Hsiao et al., 2012). Hence, conditions that promote the process of mitosis will interrupt ciliogenesis.

Although the cell cycle influences ciliogenesis, the expression of cilia is more dependent on the culture conditions. Lim et al. reported that ciliary incidence was negatively related to fetal bovine serum because primary cilia reabsorb in the early stage of mitosis and reassemble during the cell cycle (Lim et al., 2015). Therefore, cells in high density or maintained in low supplemental culture media tend to be differentiated and thus have an increased incidence of ciliogenesis.

## Ciliary Structure

The cilium is an isolated, membrane-covered, and rod-liked organelle that emanates from the surface of stagnated or differentiated eukaryotic cells. Generally, the diameter of a cilium is about 0.2 μm, and its length varies a lot (Pala et al., 2018). Cilia on the cultured renal cells may extend to more than 50 μm into the culture medium (Roth et al., 1988). However, *in vivo* study, Kim et al. reported that the ciliary length is approximately 1–2 μm in mice (van der Heiden et al., 2008).

**FIGURE 1:**
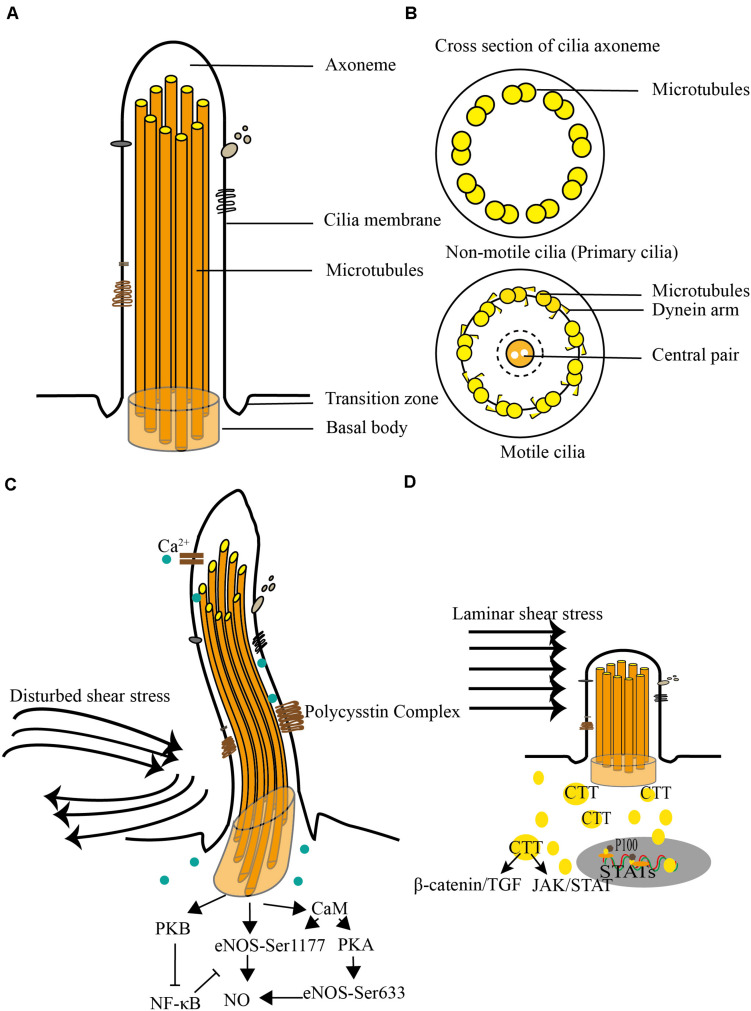
Structure and degradation of a cilium under shear stress. **(A)** A cilium consists ciliary membrane, axoneme, and basal body. There is a transition zone between the ciliary membrane and cell membrane. **(B)** Cross section of a cilium. The axoneme has 9 + 0 or 9 + 2 microtubules. Compare with motile cilia, the non-motile cilium is short of dynein arms and radical spokes and is termed as primary cilia. **(C)** Disturbed shear stress triggered Ca^2+^ influx which results in endothelial nitric oxide synthase activation and NO production. **(D)** Primary cilia become to be shorter and ultimately degraded under laminar shear stress.

A cilium is made up of the membrane, axoneme, and basal body (Figures 1A,B). The ciliary membrane is a kind of lipid bilayer that extrudes from the cell surface and is concentrated with ion channels and receptors, such as the calcium channel, somatostatin receptor, platelet-derived growth factor receptor, and melanin hormone receptor (Christensen et al., 2007). The axoneme, the core structure of a cilium, is composed of microtubules, including α-tubulin and β-tubulin. Cilia can be categorized into motile and non-motile cilia based on the different structure of the axoneme. A motile cilium contains nine peripheral doublet microtubules and a central pair of microtubules (9 + 2). Non-motile cilium contains nine double microtubules and does not have the central pair of microtubules (9 + 0) (Feistel and Blum, 2006; Salathe, 2007). A motile cilium also contains dynein arms, radial spokes, and a central pair of projections that regulate motility or generate power for ciliary motility (Figure 1B). Non-motile cilia are also known as primary cilia. The main difference with motile cilium is that the primary cilium is absent of the central pair of microtubules, the dynein arms, and radials. The basal body is derived from the distal end of the centriole and serves as a cornerstone for the ciliary axoneme. Except for its anchoring function, the basal body also specializes in regulating material transportation between the ciliary compartment and cytoplasm (Pazour et al., 2000; Hsiao et al., 2012).

The stability of the axoneme is partly maintained by the intraflagellar transport (IFT) complex. The cilium is a micro-organelle and not available to synthesize proteins. IFT complex B transports vesicles that contain proteins and micro-RNAs into the cilium, and IFT complex A ships vesicles out of the cilium. A balance between the anterograde and retrograde material transporting stabilizes the ciliary structure, and a disruption of the balance will result in the collapse of a cilium (Hsiao et al., 2012).

## Ciliary Function

Primary cilia mainly function as sensory organelles. In the retina, ciliated cells receive and transduce light stimuli, while the cells in olfactory organs are believed to sense and transfer odorant (Richardson, 1969; Glees and Spoerri, 1978). Defects in the primary cilia could impair the transport of photoreceptor transduction proteins and ultimately result in blindness (Insinna and Besharse, 2008). Likewise, the dysfunction of olfactory cilia leads to anosmia (Kulaga et al., 2004). In addition to sense, the stimuli of light or odor, ciliated cells in other organs, such as the brain, heart, liver, pancreas, kidney, and oviduct, are thought to sense mechanical, chemical, and osmotic changes (Pazour and Witman, 2003; Christensen et al., 2007). Moreover, cilia also take charge of material transportation. Cilia protrude from the cell membrane, are connected with the external environment, and absorb the necessary materials for cellular metabolism.

Ciliary function correlates with its morphological features based on the length and bending of a cilium. The polymerization of α-tubulin and β-tubulin sustain the stability of the cilium. Posttranslational modifications of tubulin are evidenced to regulate ciliary assembly. Acetylation and glycosylation stabilize tubulin polymeride and promote the extending of a cilium, and glutamylation disassembles cilia via destabilizing tubulin (Gadadhar et al., 2017; Magiera et al., 2018; Ki et al., 2020). Histone deacetylase six was reported to degrade cilia via deacetylating tubulin (Sanchez et al., 2014). The vesicles affiliated with the axoneme also helped maintain stability. It was also reported that Rab7, Rab23, and Rab35 regulated vesicle trafficking could promote the formation and composition of cilia (Lim and Tang, 2015; Kuhns et al., 2019; Wang et al., 2019). Several other factors influence ciliary lengths, such as rapamycin (Sherpa et al., 2016), pH (Atkinson et al., 2019), hypoxia (Verghese et al., 2011), lithium (Wilson and Lefebvre, 2004), Gd^3+^ or forskolin (Besschetnova et al., 2010), cytochalasin D (Cooper and Schafer, 2000), jasplakinolide (Sharma et al., 2011), and dopamine (Abdul-Majeed and Nauli, 2011).

## Cilia in the Cardiovascular System

Primary cilia are blood flow sensors that can sense shear stress as low as at least 0.007 dynes/cm^2^ (Haycraft et al., 2007). Numerous studies have reported the presence of ciliated cells in the embryonic heart. Kim et al. analyzed ciliary distribution in the embryonic cardiac cavity of chickens. They found that primary cilia are restricted to low and oscillatory shear stress regions in the embryonic heart (van der Heiden et al., 2006). In contrast to the atrioventricular canal, increased cilia can be found in the atrium and distal of the aortic valve, where blood flow is disturbed. While in the endocardial cushions, where shear stress is high, the focal cells are non-ciliated (van der Heiden et al., 2006; Kaur et al., 2018). Recently, Sarbjot et al. reported ciliated cells in embryonic, neonatal, and young rat hearts, and they found the ciliated cells non-exclusively protrude from fibroblasts (Villalobos et al., 2019). These studies suggest that primary cilia are involved in embryonic heart development. In contrast, primary cilia in young or adult heart play roles in repairing cardiomyocytes following ischemia/reperfusion injury and myocardial infarction.

**FIGURE 2:**
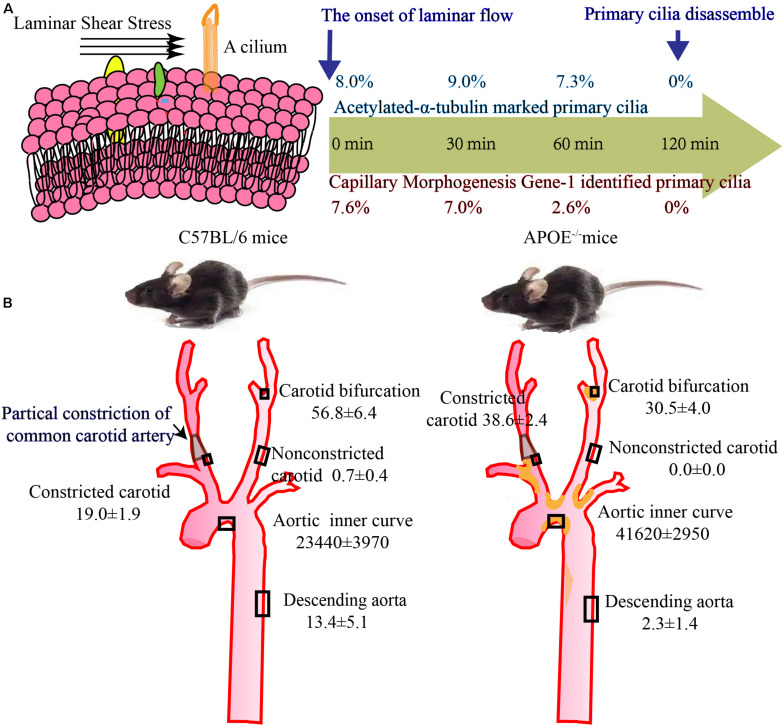
Number of cilia *in vitro* and *in vivo* study. **(A)** The incidence of primary cilia is about 8% in static cultured ECs, gradually decreased with laminar flow and drop to 0% upon 2 h flow stimulation (Iomini et al., 2004). **(B)** Quantification of primary cilia per 0.005 mm^3^ in carotid and 0.5 mm^3^ in aorta. In WT and ApoE^–/–^ mice, aorta and carotids were serially sectioned at 5 μm and then mounted to construct 3D image. Acetylated α tubulin was used to stain primary cilia and numbers of cilia per 0.005 mm^3^ in carotid and 0.5 mm^3^ in aorta are counted. Primary cilia are accumulated aortic arch, bifurcations, and constricted carotids, however, can nearly be detected in non-constricted carotids and descending aorta (van der Heiden et al., 2008).

In artery tree, primary cilia are enriched at branches, bifurcations, and inner curves, where blood flow is disturbed. In straight vessels, where shear stress is laminar, cilia are short and sparse. Carlo et al. detected ciliary expression in human umbilical vein endothelial cells treated with a laminar shear stress of 15 dynes/cm^2^ for different time lengths (Iomini et al., 2004). The incidence of primary cilia decreased with the extension of shear time, and almost no cilia can be detected when cells are exposed to laminar flow for more than 2 h (Figure 2A). In an *in vivo* study, Kim et al. analyzed ciliated cells in some specific sites in wild type and ApoE^–/–^ mice, and in these mice, the carotid flow pattern was changed by the placement of a carotid constriction cast (van der Heiden et al., 2008). Compared with laminar shear stress regions, such as the outer curve and descending aorta, primary cilia are more available in areas with low and oscillatory shear stress, including the inner arch and bifurcations. As for carotid cast modified regions, primary cilia are enriched in proximal and distal sites of the cast where shear stress is disturbed; however, they are absent in cast regions, where shear stress is high (Figure 2B). Colin et al. detected ciliated ECs in the mouse aorta. They found that cilia were more common on the inner curvature, with an approximate incidence of 28% in the aortic arch and only about 3% on the outer curvature (Dinsmore and Reiter, 2016).

The methodological difference may contribute to the varied incidence of ciliated cells in the artery. However, these results do not affect the fact that ciliated cells are concentrated in disturbed shear stress regions.

The exact mechanism of the non-uniform distribution of cilia in the artery tree remains unknown. Some researchers proposed that primary cilia cannot stand against a high level of flow, which will disassemble cilia. Once cilia are disassembled, IFT facilitates the transfer of ciliary membrane-located receptors, ion channels, and signal transduction elements into the ciliary flagella and then recruited to the cytoplasm to control cellular processes.

## Cilia and Atherosclerosis

Cilia are sensitive to shear force and their presence in the artery is positively associated with atherosclerosis plaques. A previous study reported that primary cilia promote atherosclerosis. The authors analyzed the distribution of plaques and primary cilia and found a positive connection between atherosclerosis and primary cilia. They concluded that primary cilia accelerate the progress of atherosclerosis (van der Heiden et al., 2008). On the contrary, some other studies reported that primary cilia inhibit atherosclerosis. Egorova et al. (2011) found that primary cilia attenuate vascular calcification in mice. Gonzalo and colleagues further explored the mechanism and suggested that no-ciliated ECs are prone to endothelial-to-mesenchymal transition via activating transcription factor SLUG (Sanchez-Duffhues et al., 2015). The other evidence was from Dinsmore and colleagues. They found that Tek-Cre IFT88^C/–^ ApoE^–/–^ mice (Tek-Cre IFT88^C/–^ mice are deprived of primary cilia) developed a more severe lesion area than Tek-Cre IFT88^C/+^ ApoE^–/–^ mice (Dinsmore and Reiter, 2016). Although the mechanism is not clear, Bernhard and Thomas had interesting findings (Schermer and Benzing, 2016). The controversial conclusions of Kim et al. were not convincing because they were based on the idea that cilia have a similar distribution as plaques in the artery. Studies that explore the effects of cilia on atherosclerosis are limited, and we believe that cilia protect endothelial function against the disturbed flow and inhibit atherosclerosis. This hypothesis is based on two points: (1) In *in vitro* studies, primary cilia have been identified to trigger Ca^2+^ influx, activate endothelial nitric oxide synthase and result in NO production (Praetorius and Spring, 2001; Hierck et al., 2008). (2) In an *in vivo* study, Dinsmore et al. confirmed the ciliary protective function via IFT88 gene modified mice. In conclusion, primary cilia are protective elements that infrequently exist in disturbed flow areas and can inhibit the progress of atherosclerosis.

## Ciliary Proteins Involved in Shear Stress

Along with their mechanosensory function, the primary cilia house numerous receptors, ion channels, transport proteins, and other protein complexes. Among these proteins, polycystin, and polaris are the most widely studied.

Polycystin, the protein product of PKD, is concentrated in cilia. Polycystin has two homologous proteins, termed polycystin 1 and polycystin 2. Polycystin 1, a transmembrane protein, and consists of N terminal domains, 11 transmembrane domains, and a small intracellular carboxy terminus. The extracellular region harbors a number of adhesion domains, and the intracellular domain is easy to undergo proteolysis (Chapin et al., 2010; Su et al., 2015). In the autosomal domain, polycystic kidney disease (ADPKD) kidney cells, the intracellular carboxy terminus of polycystin 1, are released from the membrane and translocated into the nucleus before activating STAT-dependent gene expression. Other studies have identified that the intracellular carboxy terminus of polycystin 1 harbor a JAK2 binding site and that the polycystin 1-JAK2 complex activates STAT3 (Low et al., 2006; Talbot et al., 2011). Another mechanism for shear stress-induced polycystin 1 activation occurs when polycystin 1 binds with heterotrimeric Gαi/o proteins, which, in turn, trigger the Ca^2+^ and K^+^ channels (Delmas et al., 2002).

More importantly, polycystin 1 combines with polycystin 2 and then forms a mechanosensory complex (Bertuccio et al., 2009; Su et al., 2015). Polycystin 2 is a member of the transient receptor potential (TRP) ion channel family. The intracellular carboxy domain houses an EF-hand structure that is considered to bind Ca^2+^, and studies have also shown that polycystin 2 is indispensable for Ca^2+^ influx (Nauli et al., 2003; Jin et al., 2014). It has been proposed that the polycystin complex is a signaling transduction element, in which polycystin 1 acts as a receptor for intercellular or cell matrix interactions, while polycystin 2 combines with TRP to regulate the ion channel (AbouAlaiwi et al., 2009). Moreover, polycystin 2 is also an endoplasmic reticulum located protein that regulates Ca^2+^ release from the endoplasmic reticulum (Koulen et al., 2002). However, there are some queries about whether polycystin 2 regulates Ca^2+^ influx (Delling et al., 2016). Therefore, it is uncertain whether primary cilia can trigger Ca^2+^ responses. Recently, Walker and colleagues reported that the integration of polycystin 2 into the cilia is necessary for Ca^2+^ influx (Walker et al., 2019). These studies suggest that ciliary localization of polycystin 2 is necessary for ion transport.

Polaris, encoded by IFT88, is a significant component in material transport. IFT is a transfer operation in which proteins rafts, receptors, vesicles, and ions are transported to the ciliary tip along the flagella (Kozminski et al., 1993). The vital function of IFT in maintaining primary cilia has been verified by using IFT mutants. For instance, IFT88^–/–^ mice are present in malformed or absent primary cilia and develop situs inversus, hepatic fibrosis, and polycystic kidney disease (Pazour et al., 2000).

## Ciliary Signal Transduction Involved in Shear Stress

Disturbed flow-induced ECs dysfunction is associated with an accumulated ROS, decreased NO, imbalanced proliferation and apoptosis, high turnover, increased permeability, and migration. To our best knowledge, primary cilia present in disturbed flow regions to protect cells from low or oscillatory shear stress-induced injury.

Primary cilia are involved in a variety of signal transduction pathways, such as Hedgehog (Hh) signaling (Huangfu et al., 2003), Wnt signaling (Oishi et al., 2006), growth factor signaling (Schneider et al., 2005), G protein-coupled receptor signaling (Berbari et al., 2008), and receptor tyrosine kinase signaling (Christensen et al., 2012). The canonical signaling for primary cilia-mediated cellular processes is Hh signaling. Patched-1 is a Hh receptor that localizes at the distal end of the primary cilia and suppresses activation of smoothened. Upon Hh stimulation, smoothened is released from patched-1, translocated to the cilia, and this process is indispensable for the activation of Hh-mediated downstream signaling (Corbit et al., 2005; Rohatgi et al., 2007). However, a study from Colin et al. suggested that Hh signaling might not be essential for ciliary-involved vascular development (Dinsmore and Reiter, 2016). Nauli reported that the mechanosensory function of primary cilia depends on polycystin 1 and polycystin 2 (Nauli et al., 2003). Polycystin 2 depends on functional polycystin-1, and polycystin-2^–/–^ cells do not respond to fluid shear stress. Calcium is an important second messenger to transduce fluid flow stimuli into intracellular and activates endothelial nitric oxide synthase (eNOS), promoting NO release through polycystin 2 (Koulen et al., 2002). Upon sensing shear stress, polycystin-2 triggers extracellular Ca^2+^ influx which binds to calmodulin and results in the activation of PKB and PKC (Figures 1C,D). PKB and PKC phosphorylate their downstream eNOS at the serine 1,177 and 633 sites, respectively, initiating an immediate NO synthesis (AbouAlaiwi et al., 2009). The polycystin complex is an important component in primary cilia. In cultured ECs, cilia are disrupted upon HSS stimuli. Mechanically speaking, disturbed shear stress triggers the combination of polycystin 1 and polycystin 2 and thus results in calcium influx. However, laminar shear stress disrupts polycystin 1, which results in the disassembly of cilia (Nauli et al., 2008; Goetz et al., 2014). The disassembly of cilia coincides with the acetylation of microtubules and the organization of microfilaments, which are involved in rearranging the cytoskeleton. Thomas et al. reported that heat shock protein 27-dependent actin organization and focal adhesion formation might contribute to cytoskeletal rearrangement (Jones et al., 2012). The disassembly of cilia also increases the expression of several proinflammation genes and cytokines, including interleukin-1, interleukin-6, transforming growth factor (TGF)-β, tumor necrosis factor-α, and nuclear factor-κB and vascular cell adhesion molecule and E-selectin (Dinsmore and Reiter, 2016).

Shear stress accelerates ciliary bending speed, and the ciliary tip touches the cell membrane and serves as an amplification of extracellular stimuli. In the absence of ciliary bending, polycystin 1 underwent hydrolysis, and its carboxyl terminal was released, translocated to the nucleus, and combined with p100 or STAT6 to activate transcriptional activity in cystic disease (Low et al., 2006).

In the heart cavity, non-ciliated ECs prefer to undergo an endothelial-to-mesenchymal transition and calcification. In this process, ECs possess a mesenchymal phenotype and migrate into the cardiac jelly to form the rudiment of cardiac valves. Egorova et al. (2011) found that primary cilia are indispensable in endothelial-to-mesenchymal transition via a TGFβ/ALK5-dependent pathway.

## Conclusion and Perspective

Primary cilia are not uniformly distributed in artery trees. They are concentrated in disturbed flow regions, such as the bifurcations and inner curves, and are barely detected in straight areas. Animal studies have confirmed that primary cilia protect ECs against disturbed flow induced injury and inhibit atherosclerosis; however, the mechanism is not clear. Considering ciliary distribution in the artery tree, two questions need to be clarified: why cilia are enriched in disturbed flow regions and how cilia can attenuate atherosclerosis. In this review, we discussed ciliogenesis and ciliary function in detail and want to elaborate that shear-regulated cell cycle, posttranscription of tubulin, and accelerated vesicles trafficking might be involved in the process.

Further studies exploring how cilia senses and transduces mechanical stimuli will develop understanding of its protective effects in repairing disturbed flow-induced cellular damage. However, there are no effective pharmacological agents that target primary cilia specifically. The protection of primary cilia might be an important strategy in protecting endothelial cells against disturbed shear stress-induced cellular damage. In the future, more studies in this area are warranted to clarify these questions and promote clinical translation.

## Author Contributions

Z-MW wrote the manuscript. X-FG draw schematic diagrams. J-JZ and S-LC proposed the idea and revised the manuscript. All authors approved the manuscript for publication.

## Conflict of Interest

The authors declare that the research was conducted in the absence of any commercial or financial relationships that could be construed as a potential conflict of interest.
